# Natural hazard triggered technological risks in the Yangtze River Economic Belt, China

**DOI:** 10.1038/s41598-021-93353-y

**Published:** 2021-07-05

**Authors:** Yue Gao, Guozhi Cao, Ping Ni, Yue Tang, Yetong Liu, Jun Bi, Zongwei Ma

**Affiliations:** 1 State Key Laboratory of Pollution Control and Resource Reuse, School of the Environment, Nanjing University, 163 Xianlin Avenue, Nanjing, 210023 China; 2 Jiangsu Collaborative Innovation Center of Atmospheric Environment and Equipment Technology (CICAEET), Nanjing University of Information Science and Technology, Nanjing, Jiangsu China; 3 State Environmental Protection Key Laboratory of Environmental Planning and Policy Simulation, Chinese Academy for Environmental Planning, Beijing, China

**Keywords:** Environmental sciences, Natural hazards

## Abstract

With the continuous occurrence of natural disasters, natural hazard triggered technological accident (Natech) risks also follow. At present, many countries have performed much research on Natech risks. However, there is still a lack of Natech research at the regional or watershed level in China. The Yangtze River Economic Belt (YREB) is not only an industrially intensive development area but also an area with frequent natural disasters. In this study, we selected the YREB as a typical case to study the Natech risk triggered by floods, geological disasters, and typhoons at the regional or watershed level. Four types of risk indicators representing risk sources, natural hazard factors, control levels, and vulnerabilities were developed to assess the spatial patterns of the Natech risks of the YREB. The results show that the Natech risk triggered by floods and typhoons is more serious in eastern area and central area than in western zone and that the Natech risk triggered by geological disasters is more serious in the west part. Approximately 7.85% of the areas are at relatively high-risk and above the Natech risk level based on the comprehensive assessment of three types of Natech risks. The combined population of these areas accounts for approximately 15.67% of the whole YREB, and the combined GDP accounts for approximately 25.41%. It can be predicted that the occurrence of Natech risks in these areas will cause serious harm to both the people and the economy. This work will provide the basis and key management direction for Natech risk management in the YREB.

## Introduction

With continuous industrialization, heavy industry has become one of the leading industrial sectors in China. China is one of the countries in the world that suffers from severe natural disasters. The environmental and safety incidents of industrial enterprises triggered by natural disasters have become a primary concern for authorities. This kind of incident triggered by natural disasters is called a natural hazard triggered technological accident (Natech), which was first proposed by Showalter and Myers^1^. Natech is a kind of event with low probability but high impact and may involve the release of hazardous materials into the environment and further result in extensive pollution in the region^2^. For example, Hurricanes Katrina and Rita in the United States caused a large amount of crude oil leakage and diffusion in the storage tank of a factory^3^. In 2011, the geological disaster and tsunami affected Japan's industrial facilities, industrial parks, and port terminals^4^. Natech incidents triggered by floods in Central Europe resulted in chlorine being released into the air in large quantities, and a large number of hazardous chemicals were leaked into the water^5^. In addition, typical cases have been conducted on the Natech effects of the 1999 Kocaeli geological disaster in Turkey, the Wenchuan geological disaster in China and Hurricane Harvey in 2017^6–8^. Due to its frequent occurrences and high impact, Natech risk has become an important research focus globally^9^.

Compared with Natech risk research, many researchers have made many contributions to natural disaster risk and technological risk. For example, Yu, et al. proposed an evaluation model to make risk assessments of four main disasters^10^. Sun et al. established a flood disaster risk analysis model to evaluate the flood disaster risk in Shanghai, Jiangsu etc^11^. Zou, et al. developed a quantitative method for the regional risk assessment of debris flows by analyzing the in-depth relations among hazard-forming environments, disaster factors and elements at risk^12^. Chen, et al. proposed a new multicriteria decision-making method to evaluate the natural disaster risk of China at the regional scale^13^. In addition, previous studies have made some contributions to regional technical risk assessments. Many researchers have performed a great deal of work on risk assessments of environmental incidents in China. The existing studies primarily focus on the enterprise^14–16^ or regional scales^17–19^.

Compared with the development of natural disasters and technological risk, many papers have also been published to address Natech risk assessment. For example, Di Franco and Salvatori summarized the state-of-the-art techniques on how Earth observation (EO) data can be useful in managing all phases of an industrial/Natech disaster^20^. Naderpour, et al. used the spatial parameters of a forest fire to model, predict, and evaluate the Natech risk triggered by fire^21^. Yang, et al. proposed an assessment framework of comprehensive water pollution by combining water quality indices and geological disaster damage indices^22^. Han, et al. developed an indicator system of Natech environmental risk assessment through an analytic hierarchy process and fuzzy evaluation model^23^.

Except for the above methodology of Natech risk assessment, some researchers consider Natech risk management and the identification of risky areas in mitigating the risks faced by an industry. For example, Cao et al. summarized the characteristics and laws of environmental emergency risk in China. In their work, the causes of the failure of risk control mechanisms, including natural factors, were analyzed^24^. In addition, a combination of statistical data and a mathematical model was used to analyze the degrees of influence of different network nodes on the system disturbance value^25^. Du et al. used spatial statistical methods and geographically weighted regression models to study the temporal and spatial evolution trends of environmental events^26^.

**Figure 1:**
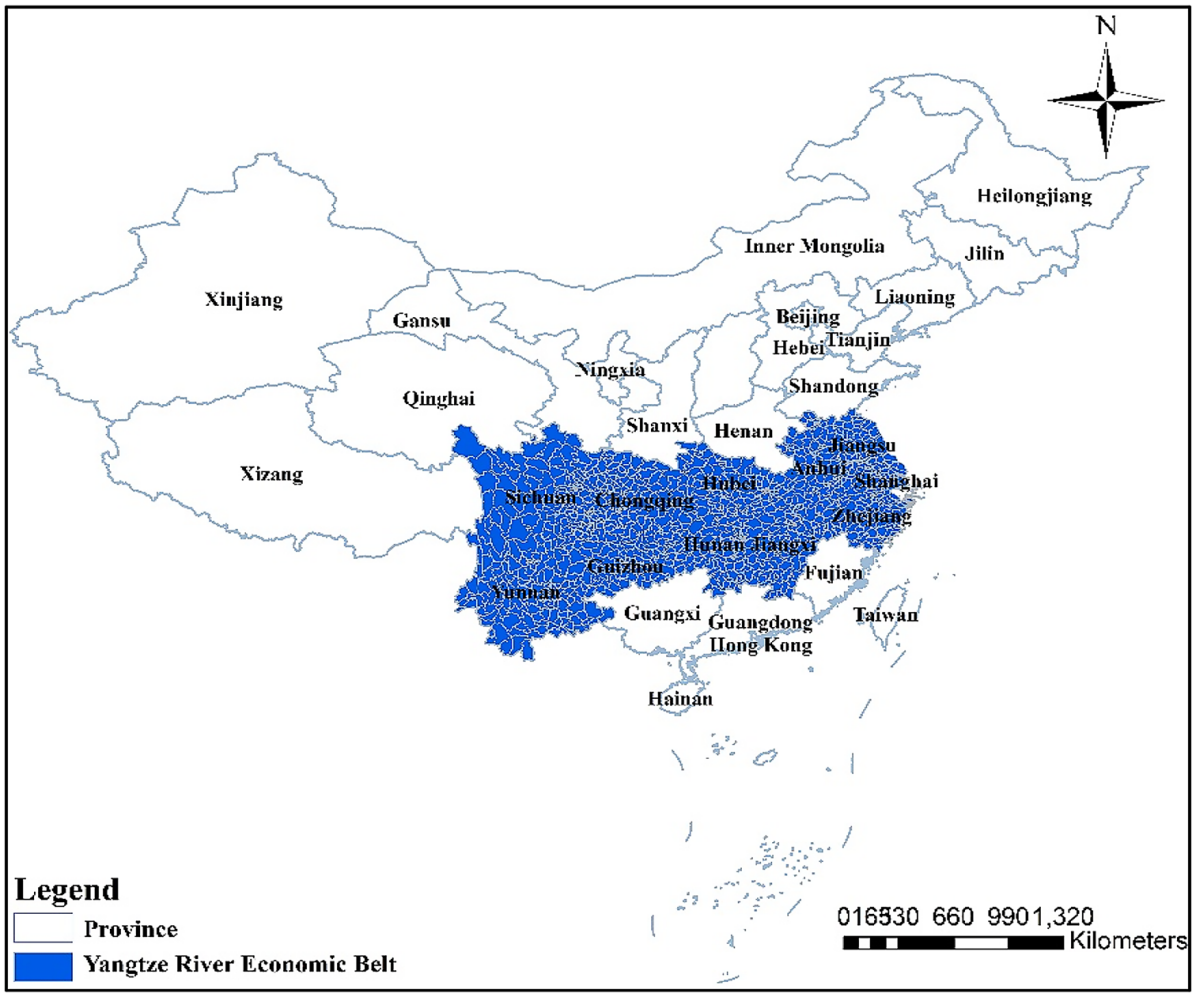
Location of the Yangtze River Economic Belt in China. Map is produced using ArcGIS 10.2 (http://www.esri.com/software/arcgis/arcgis-for-desktop).

It can be seen that the research on Natech risk has mostly focused on small-scale regions, such as enterprise, an industrial park or a province. Most of the studies have focused on the identification of key nodes for risk failure and the direct consequences of such risk. However, China's current research on the evaluation of Natech risk at the regional level is still scarce. In this paper, the Yangtze River Economic Belt (YREB) (Fig. 1) is selected as a typical area of Natech risk research. On the one hand, the YREB is a concentrated distribution area of China's industrial parks. There are more than 12,000 enterprises, mainly including chemical, pharmaceutical, and other key industries^27^. The GDP of the area is more than 40% of the whole country^28^. On the other hand, the YREB is a region with frequent natural disasters. Geological and geomorphological disasters are prominent in the upper reaches, hydrometeorological disasters are evident in the central and lower reaches. For example, about 37% of the major geological disasters in China are developed in YREB. In particular, the upper reaches of Chongqing, Sichuan, Yunnan, and Guizhou are the areas with high incidence of large-scale geological disasters^29^. Moreover, the YREB has experienced seven massive floods (in 1860, 1870, 1931, 1935, 1954, 1998, and 2010)^30^ etc. According to statistics from the Ministry of Environmental Protection in 2006–2015, environmental emergencies in the YREB caused by production safety, enterprise pollution, traffic accidents, and natural disasters accounted for 44%, 59%, 47%, and 55% of the corresponding environmental emergencies in the country^31^. Once Natech events occur, a disaster chain will often be formed, which will have an extensive impact on and cause risk loss for the whole region.

The contribution of this work can be summarized as follows. (1) An index system is developed for regional Natech risk assessment, which can be used to quantify the impacts of Natech risk on the economic, environmental and social aspects. (2) The Natech risk triggered by natural disasters, such as floods, geological disasters, and typhoons, is evaluated; the findings can assist decision makers in making effective policies for risk management. (3) The framework of this work provides a reference for risk assessment at the macro level.

## Data and method

### Data

#### Risk sources of industrial enterprises

The basic information of more than 140 thousand industrial enterprises in China comes from the China Environmental Statistics Database (ESD) for 2015^32^. The risk sources collected information included the codes, names, longitudes and latitudes, scales of industrial enterprises and the industrial sectors to which the enterprises belong. Then the Q value is collected. It is defined as the ratio of the risky substance quantity that was used and stored in the enterprise to the threshold quantity relating to the physical toxicity, environmental hazard, and diffusion characteristics of the substance^33^. The Q value is a significant impact variable in the occurrence of an accident^35^. If an enterprise has more than one risk substance, the Q value equals $\sum _{n=1}^{m}{Q}_{n}$, where ${Q}_{n}$ is the sub Q value of risk substance n^34,35^.

The final Q value of each chemical enterprise is collected and determined mainly through the following channels. First of all, the data is collected from of the national investigation on environmental risks and chemicals from enterprises in key industrial sectors, which was conducted in 2010. Secondly, the data is collected from of the investigation on environmental incident risks among enterprises in Jiangsu Province in 2015. Third, the data is collected from of investigations on enterprise environmental risks in some regions such as Urumqi City (2015), Tianjin Binhai New District (2015), etc. The Q value is obtained through the above channels. And the Q values are assumed that did not change between the investigation years and assessment year. For the enterprises not included in these investigations, their Q values indirectly are obtained by using the enterprises’ scale and sector information from ESD of 2015. That is, the Q value of other enterprises is taken as the average of the Q value of existing enterprises in the same industry with the same enterprise scale^36,37^. Therefore, the Q values can represent the intensity of risk sources. This Q value dataset is applied to assess the risks of environmental incidents in the YREB^36^ as well as throughout China^37^.

#### Hazard data on natural disasters

The hazard data on natural disaster risk sources mainly come from remote-sensing data and basic geographic data, including flood, geological disaster and typhoon disaster data.

The flood hazard factor data are collected from the following sources. (1) Flood inundation range data of the YREB in 1998, 2002, 2006, and 2010 are obtained by Landsat and Modis satellite image interpretation. (2) Digital elevation data mainly come from the Environment Data Cloud Platform of the Chinese Academy of Science (EDCP-CAS, http://www.resdc.cn/DataList.aspx). (3) The primary site coordinates, flood level, peak discharge, and flood return period during a flood, are mainly derived from the annual report of the Ministry of Water Resources (http://www.mwr.gov.cn/sj/) and site information of the China Water Station.

The geological disasters factor data are collected from the following sources. (1) The data on earthquake points in China and surrounding areas since 1900 mainly come from the EDCP-CAS (http://www.resdc.cn/data.aspx?DATAID=296). The data mainly include important information such as the longitude and latitude coordinates, occurrence time, magnitude, focal depth, and location. (2) The data of seismic intensity caused by geological disasters come from the EDCP-CAS (http://www.resdc.cn/data.aspx?DATAID=290). The data includes 7 major types of geological disasters: collapse, subsidence, debris flow, ground subsidence, ground fissures, landslides, and slopes. (3) The geological deformation data came from the European Space Agency (ESA) sentinel online (https://scihub.copernicus.eu/dhus/#/home).

The typhoon hazard factor data are collected from the following sources. (1) The data on typhoon tracks in the Western Pacific come from EDCP-CAS (http://www.resdc.cn/data.aspx?DATAID=293). The data mainly include important information such as the track points and paths of 1782 typhoons from 1951 to 2018 and the maximum wind force. (2) The precipitation data affected during the typhoon are derived from the precipitation station data of the China Meteorological Administration from 2000 to 2018. (http://www.resdc.cn/data.aspx?DATAID=282).

#### Other data

Control mechanism data. (1) The data on the proportion of investment in regional environmental management come from the “China Environmental Statistics Yearbook” from 2010 to 2017. (2) The original data on enterprise environmental irregularities come from the “Institute of Public & Environmental Affairs” (IPE, http://www.ipe.org.cn/index.html). (3) Statistics on the number of emergencies between 2010 and 2018 come from “Annual Statistic Report on Environment in China”. (4) Data on energy conservation and environmental protection expenditure comes from provincial statistical yearbooks and government websites.

Vulnerability data. (1) The population data in 2015 come from EDCP-CAS. (2) The GDP data per unit area of each district or county come from the Statistical Yearbook of 11 provinces and cities in the YREB. (3) The vector data on the national water attribute come from the “National Geomatics Center of China” (http://www.webmap.cn/main.do?method=index). (4) The sensitive point vector data of education and medical treatment come from the EDCP-CAS (http://www.resdc.cn/data.aspx?DATAID=330).

### Construction of indicator system

**Figure 2:**
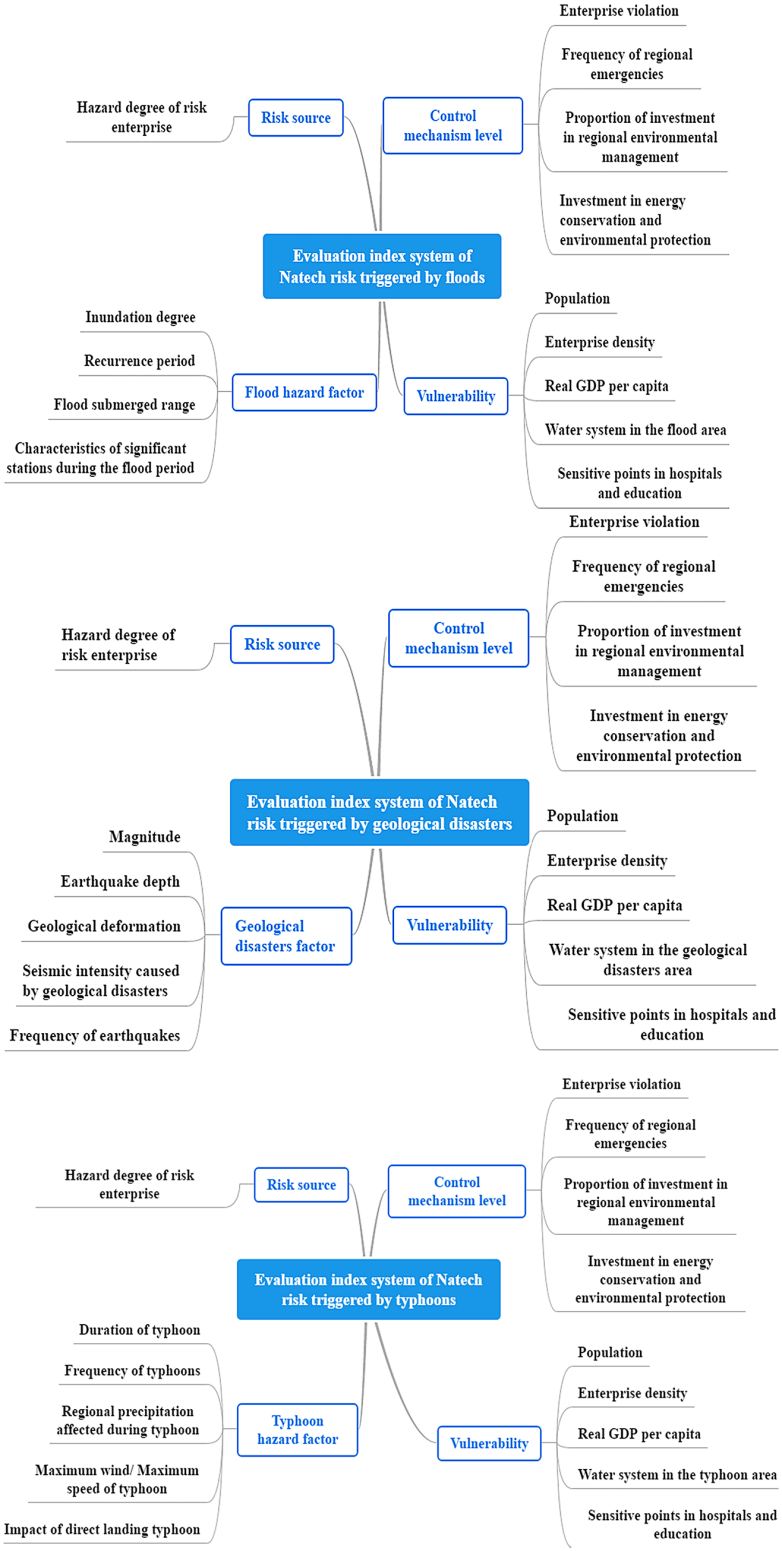
The evaluation index system of Natech risk in the YREB.

The indicator system was constructed considering four aspects: the risk source, control level, natural disaster hazard factors, and vulnerability. These indicators are shown in Fig. 2.

### Calculation of Natech risk indices and determination of index weight

The calculations of the risk indices of Natech risks triggered by floods, geological disasters, and typhoons are described as follows. We used the analytical hierarchy process (AHP)^38^ to calculate the weights of the indicators. The pair-wise comparison matrix is shown in Table S1–S5 in the Supplementary Materials (SM). The relative weight of each index is shown in Tables 1, 2 and 3, and the consistency checking result (CR < 0.1) indicates each index’s reasonability and effectivity.

**Table 1:** The assessment system of Natech risk triggered by floods.

Target layer	Evaluation index	Indicator description and classification basis	Grading standards	Weights	Score
Risk source indicators (SF)	Hazard degree of risk enterprise	Hazard degree of risk enterprise at different Q levels of districts or counties (%). Classification according to the Eq. (2)	Equation (2)	1	100
Hazard factor indicators of flood (HF)	Flood submerged range (F1)	The proportion of each district or county flood inundation in the total inundation (%). Classification according to the part of “Flood submerged range and inundation risk level”	The total inundation frequency of each district or county/4	0.3204	100
	Inundation degree (F2)	Superimpose DEM data on submerged area data to classify submerged levels. Based on the statistical results of the sample data is sorted from small to large, grades are set based on the 5, 15, 50, 85 and 95 percentiles of all samples	Level 1	0.453	90
			Level 2		75
			Level 3		60
			Level 4		45
			Level 5		30
			Level 6		15
	Characteristics of significant stations during the flood period (F3)	The over-alarming situation of the highest water level of the flood peak at the main stations (m). Classification based on the statistical analysis results over the years	< 0.5	0.1405	20
			[0.5,1.5)		40
			[1.5,2.5)		60
			[2.5,3.5)		80
			≥ 3.5		100
	Recurrence period (F4)	Recurrence period of significant floods in district or county (year). Classification according to the return period of the historical flood	< 10	0.0861	25
			[10,20)		50
			[20,50)		75
			≥ 50		100
Control mechanism level indicators (CF)	Enterprise violation (C1)	The proportion of enterprises with violation records in the administrative area in the total number of enterprises (%). Based on the statistical results of the sample data is sorted from small to large, grades are set based on the 10, 50 and 90 percentiles of all samples	< 2%	0.4203	25
			[2%, 10.5%)		50
			[10.5%, 35%)		75
			≥ 35%		100
	Proportion of investment in regional environmental management (C2)	The proportion of environmental pollution control investment in GDP of administrative regions from 2010 to 2017 (%). Based on the statistical results of the sample data is sorted from small to large, grades are set based on 10, 50 and 95 percentiles of all samples	< 0.78%	0.1899	100
			[0.783%, 1.15%)		75
			[1.15%, 1.8%)		50
			≥ 1.8%		25
	Frequency of regional emergencies (C3)	The proportion of the frequency of emergencies in the administrative region in the total number of regional emergencies from 2009 to 2018 (%). Classification according to statistical results. Based on the statistical results of the sample data is sorted from small to large, grades are set based on the 10, 50 and 90 percentiles of all samples	< 5%	0.1213	25
			[5%, 7.5%)		50
			[7.5%, 15%)		75
			≥ 15%		100
	Investment in energy conservation and environmental protection (C4)	The proportion of energy conservation and environmental protection investment in total expenditure (%). Based on the statistical results of the sample data is sorted from small to large, grades are set based on the 10, 55 and 95 percentiles of all samples	< 1.6%	0.2685	100
			[1.6%, 2.6%)		75
			[2.6%, 4%)		50
			≥ 4%		25
Vulnerability indicators (VF)	Population (V1)	The population of districts or counties. Classification according to Eq. (3)	Equation (3)	0.1737	100
	Sensitive points in hospitals and education (V2)	A number of medical and educational institutions in districts or counties. Classification according to Eq. (4)	Equation (4)	0.1737	100
	Real GDP per capita (V3)	Average per capita GDP from 2015 to 2018 (yuan). Based on the statistical results of the sample data is sorted from small to large, grades are set based on the 10, 50 and 90 percentiles of all samples	< 19,000	0.4794	25
			[19000, 39,000)		50
			[39000, 105,000)		75
			≥ 105,000		100
	Enterprise density (V4)	The proportion of the number of risk enterprises in the area of districts or counties (number/km^2^). Based on the statistical results of the sample data is sorted from small to large, grades are set based on the 10, 50 and 90 percentiles of all samples	< 0.005	0.1011	25
			[0.005, 0.03)		50
			[0.03, 0.2)		75
			≥ 0.2		100
	Water system in the flood area (V5)	Grade of target water quality flowing through districts or counties. Classification according to the classification standard of water quality	Grade 1	0.0721	100
			Grade 2		80
			Grade 3		60
			Grade 4		40
			Grade 5		20

**Table 2:** The assessment system of Natech risk triggered by geological disasters.

Target layer	Evaluation index	Indicator description and classification basis	Grading standards	Weights	Score
Risk source indicators (SG)	Hazard degree of risk enterprise	Hazard degree of risk enterprise in different Q levels of districts or counties (%). Classification according to Eq. (2)	Equation (2)	1	100
Hazard factor indicators of geological disasters (HG)	Magnitude (G1)	The degree of magnitude hazard in districts or counties (%). Classification according to the Eq. (5) and statistical data calculation results	< 1	0.2139	25
			[1, 10)		50
			[10, 50)		75
			≥ 50		100
	Earthquakes depth (G2)	The degree of earthquakes depth hazard in districts or counties (%). Classification according to the Eq. (6) and statistical data calculation results	< 1	0.1069	25
			[1, 10)		50
			[10, 50)		75
			≥ 50		100
	Seismic intensity caused by geological disasters (G3)	Average seismic intensity level in districts or counties Classification according to the China seismic intensity scale	< 5	0.3242	25
			[5, 7)		50
			[7, 9)		75
			≥ 9		100
	Frequency of earthquakes (G4)	Frequency of earthquakes in districts or counties from 1900 to 2018. Statistical analysis and classification based on the logging data of earthquakes. Based on the statistical results of the sample data is sorted from small to large, grades are set based on the 30 and 80 percentiles of all samples	[1, 2)	0.1411	30
			[2, 12)		60
			≥ 12		90
	Geological deformation (G5)	Land subsidence rate caused by geological disasters of earthquakes in districts or counties (mm/a)	Equation (8)	0.2139	100

**Table 3:** The assessment system of Natech risk triggered by typhoons.

Target layer	Evaluation index	Indicator description and classification basis	Grading standards	Weights	Score
Risk source indicators (ST)	Hazard degree of risk enterprise	Hazard degree of risk enterprise in different Q levels of districts or counties (%). Classification according to the Eq. (2)	Equation (2)	1	100
Hazard factor indicators of typhoon (HT)	Maximum wind/Maximum speed of typhoon (T1)	The wind speed and grade of typhoons in 1951–2018 were classified (grade or wind speed (m/s)). Classification according to typhoon classification standard of Central Meteorological Station	< 8 or < 17	0.1124	30
			[8, 12) or [17, 30)		60
			≥ 12 or ≥ 30		90
	Impact of direct landing typhoon (T2)	Frequency of typhoons in counties from 1951 to 2018. Based on the statistical results of the sample data is sorted from small to large, grades are set based on the 20, 60 and 90 percentiles of all samples	≤ 1	0.1745	25
			[2, 6)		50
			[6, 13)		75
			≥ 13		100
	Frequency of typhoons (T3)	Frequency of typhoons affected by districts or counties from 1951 to 2018. Based on the statistical results of the sample data is sorted from small to large, grades are set based on the 10, 50 and 90 percentiles of all samples	[1, 3)	0.2707	25
			[3, 40)		50
			[40, 180)		75
			≥ 180		100
	Regional precipitation affected during typhoon (T4)	Annual average typhoon rainfall of districts or counties from 2000 to 2018 (mm). Classification according to the 24-h rainfall classification standard of meteorology	< 25	0.3572	20
			[25, 50)		40
			[50, 100)		60
			[100, 250)		80
			≥ 250		100
	Duration of typhoon (T5)	Duration of each typhoon in districts or counties from 1951 to 2018 (days). Based on the statistical results of the sample data is sorted from small to large, grades are set based on the 10, 40 and 80 percentiles of all samples	[3.75, 8.25)	0.0852	25
			[8.25, 11.75)		50
			[11.75, 16.25)		75
			≥ 16.25		100

#### Risk index calculation of Natech risk triggered by floods

1. **Risk level classification standard of enterprise risk sources**
  The Q value and the threshold quantity of risky substances refer to the grading standard method provided by the Ministry of Ecology and Environment (MEE)^33^ and can be found in the "Classification method of for environmental accident risk of the enterprise (HJ941-2018)". The calculation formula of Q is as follows.
  1 $$\mathrm{Q}=\frac{{w}_{1}}{{W}_{1}}+\frac{{w}_{2}}{{W}_{2}}+\dots +\frac{{w}_{n}}{{W}_{n}}$$
  where the ${w}_{1},{w}_{2},\dots ,{w}_{\mathrm{n}}$ means that the stock of each risk substance, t
  The ${W}_{1}$, ${W}_{2},\dots ,{W}_{\mathrm{n}}$ means that the critical quantity of each risk substance, t
  The quantification of Q refers to the tenfold conversion relationship for environmentally hazardous substances in German inventory law. Because of the actual situation of existing data processing and the lower hierarchy of the classification method, two new levels have been added. The method is mentioned in the “enterprise environmental risk grading assessment method (preparation instruction)”^39^. As a result, the hazards of risk sources were recategorized into 6 levels according to the size of Q. The classification criteria as follows.
  Q < 1, represented by Q0.
  1 ≤ Q < 10, represented by Q1.
  10 ≤ Q < 100, represented by Q2.
  100 ≤ Q < 1000, represented by Q3.
  1000 ≤ Q < 10,000, represented by Q4.
  Q ≥ 10,000, represented by Q5.
  Among them, the risk levels from Q0 to Q5 increase in order. In addition, it is necessary to consider the degree of hazard contribution under different Q value ranges. The hazard degree of different Q-level intervals in each district is calculated. The calculation formula and classification criteria are as follows.
  $${HQ}_{\left(district\right)}=\sum _{i=1}^{n}\left({Q}_{q}\times {Cq}_{\left(hazard\right)}\right)$$
  2 $${HQ}_{(district)}^{*}=\frac{HQ-{HQ}_{min}}{{HQ}_{max}-{HQ}_{min}}\times 100$$
  where *n* is the total number of Q-level classifications in each district or country. ${HQ}_{(district)}$ represents the degree of hazard in different Q-level intervals of each district or county. ${Q}_{q}$ represents the number of enterprises in different Q intervals of the district. ${Cq}_{(hazard)}$ represents the hazard contribution in different Q intervals. ${HQ}_{(district)}^{*}$ is the standardized treatment of ${HQ}_{\left(district\right).}$ ${Cq}_{(hazard)}$ is assigned as 15%, 30%, 45%, 60%, 75%, and 90% from Q0 to Q5, respectively.
2. **Flood submerged range and inundation risk level**
  The impact of topography on flood formation is mainly reflected in the fact that the lower the terrain elevation is, the more vulnerable it is to floods. First, based on the relatively serious and representative floods in history, the floods of 1998, 2002, 2006 and 2010 were selected as the representative objects of the research. The result of the flood inundation range was determined by calculating the difference in water area between the flood period and the normal period. By calculating the proportion of each district or county flood inundation to the total inundation, the temporal and spatial impact of a flood were expressed.
  Then, combined with the elevation data of the inundated area, the inundation degree of the inundated area is obtained. Finally, the degree of inundation is divided into six grades. From levels 1 to 6, the terrain increases, and the degree of inundation decreases in turn.
3. **Vulnerability indicators of the population**
  The vulnerability index of the population is shown in Eq. (3):
  3 $${V}_{(\mathrm{p}\mathrm{o}\mathrm{p})}=\frac{{pop}_{(\mathrm{d}\mathrm{i}\mathrm{s}\mathrm{t}\mathrm{r}\mathrm{i}\mathrm{c}\mathrm{t})}-{pop}_{min}}{{pop}_{max}-{pop}_{min}}\times 100$$
  where ${V}_{(\mathrm{p}\mathrm{o}\mathrm{p})}$ indicates the vulnerability index of the population. ${pop}_{(\mathrm{d}\mathrm{i}\mathrm{s}\mathrm{t}\mathrm{r}\mathrm{i}\mathrm{c}\mathrm{t})}$ is the population of a district or a county. ${pop}_{min}$ is the minimum population of districts or counties. ${pop}_{max}$ is the maximum population of districts or counties.
4. **Vulnerability indicators of the sensitive points of medical education**

The vulnerability indicator of the sensitive points of medical education is written as Eq. (4):

4 $${V}_{(\mathrm{L}\mathrm{C}\mathrm{M}\mathrm{E})}=\frac{{\mathrm{L}\mathrm{C}\mathrm{M}\mathrm{E}}_{(\mathrm{d}\mathrm{i}\mathrm{s}\mathrm{t}\mathrm{r}\mathrm{i}\mathrm{c}\mathrm{t})}-{\mathrm{L}\mathrm{C}\mathrm{M}\mathrm{E}}_{min}}{{\mathrm{L}\mathrm{C}\mathrm{M}\mathrm{E}}_{max}-{\mathrm{L}\mathrm{C}\mathrm{M}\mathrm{E}}_{min}}\times 100$$

where ${\mathrm{V}}_{(\mathrm{L}\mathrm{C}\mathrm{M}\mathrm{E})}$ indicates the vulnerability index of medical education. ${\mathrm{L}\mathrm{C}\mathrm{M}\mathrm{E}}_{(\mathrm{d}\mathrm{i}\mathrm{s}\mathrm{t}\mathrm{r}\mathrm{i}\mathrm{c}\mathrm{t})}$ is the number of medical education institutions in a district or county. ${\mathrm{L}\mathrm{C}\mathrm{M}\mathrm{E}}_{min}$ is the minimum number of medical education institutions in districts or counties. ${\mathrm{L}\mathrm{C}\mathrm{M}\mathrm{E}}_{max}$ is the maximum number of medical education institutions in districts or counties. The details of these indicators are summarized in Table 1.

In summary, the Natech risk index triggered by floods in each district or county is calculated as follows:

5 $${CRF}_{i}=\sqrt[4]{{{SF}_{i}\times HF}_{i}\times {CF}_{i}\times {VF}_{i}}$$

where ${CRF}_{i}$ is the Natech risk index triggered by floods in the *i*th district or county. ${SF}_{i}$ is the risk source indicator of Natech risk triggered by floods in the *i*th district or county. ${HF}_{i}$ is the hazard factor indicator of Natech risk triggered by floods in the *i*th district or county. ${CF}_{i}$ is the control mechanism level indicator of Natech risk triggered by floods in the *i*th district or county. ${VF}_{i}$ is the vulnerability indicator of Natech risk triggered by floods in the *i*th district or county.

#### Risk index calculation of Natech risk triggered by geological disasters

1. **Analysis of the magnitude hazard index**
  Based on the statistical data on earthquakes that occurred from 1900 to 2018 in the YREB, magnitude and focal depth are classified. Seismic magnitude division is based on the Richter scale division standard. Furthermore, based on the divided geological disasters levels, the hazard index of geological disasters in districts or counties is calculated, and their contribution is assigned. The degree of hazard in the region is presented in Eq. (6):
  6 $${\mathrm{H}\mathrm{m}}_{\left(district\right)}=\sum _{i=1}^{n}\left({Q}_{\left(m\right)}{\times Cm}_{(hazard)}\right)$$
  where n represents the total number of magnitude-level classifications in each district or country. ${Hm}_{(district)}$ represents the degree of hazard in different magnitude intervals of each district or county. ${Q}_{(m)}$ is the number of earthquakes with different magnitudes in each district or county. ${Cm}_{(hazard)}$ represents the hazard contribution degree in different magnitude intervals.
  Based on the Richter scale, the earthquakes magnitude is divided into (0,3], (3,5], (5,7] and above level 7, and ${Cm}_{(hazard)}$ is set as 25%, 50%, 75%, and 100%, respectively.
2. **Analysis of the hazard index of earthquakes depth**
  According to the criteria of seismic focal depth, the calculation formula and classification criteria of the degree of hazard in different magnitude intervals of each district or county are shown in Eq. (7):
  7 $${\mathrm{H}\mathrm{d}}_{\left(district\right)}=\sum _{i=1}^{n}\left({Q}_{\left(d\right)}\times {Cd}_{(hazard)}\right)$$
  where n represents the total number of seismic depth level classifications in each district or country. ${Hd}_{(district)}$ represents the degree of hazard in the different seismic depth intervals of each district or county. ${Q}_{(d)}$ is the number of earthquakes with different seismic depths in each district or county. ${Cd}_{(hazard)}$ represents the hazard contribution in different seismic depth intervals. Based on the criteria of seismic focal depth, the seismic depth is divided into (0,30], (30,60], and above 60 and ${Cd}_{(hazard)}$ is set as 90%, 60%, and 30%, respectively.
3. **Analysis of the hazard index of geological deformation**
  Based on data availability and the occurrence of geological disasters from 2016 to 2020, we finally chose data from 2016, which had more geological disasters, as the representative data. The geological deformation was calculated by the DInSAR technique^40,41^. Then, based on the geological deformation results, we calculate the average rate of land subsidence. Finally, the standardized results are presented in Eq. (8):
  8 $${\mathrm{H}\mathrm{s}}_{\left(district\right)=}=\frac{{\mathrm{S}}_{(\mathrm{d}\mathrm{i}\mathrm{s}\mathrm{t}\mathrm{r}\mathrm{i}\mathrm{c}\mathrm{t})}-{\mathrm{S}}_{min}}{{\mathrm{S}}_{max}-{\mathrm{S}}_{min}}\times 100$$
  where ${\mathrm{H}\mathrm{s}}_{\left(district\right)}$ indicates the hazard index of geological deformation. ${\mathrm{S}}_{(\mathrm{d}\mathrm{i}\mathrm{s}\mathrm{t}\mathrm{r}\mathrm{i}\mathrm{c}\mathrm{t})}$ is the average rate of land subsidence in a district or county. ${\mathrm{S}}_{min}$ is the minimum number of the average rates of land subsidence in districts or counties. ${\mathrm{S}}_{max}$ is the maximum number of the average rates of land subsidence in districts or counties. The details of indicators of are shown in Table 2.
  The Natech risk index triggered by geological disasters is calculated by the following formula:
  9 $${CRG}_{i}=\sqrt[4]{{SG}_{i}\times {HG}_{i}\times {CG}_{i}\times {VG}_{i}}$$
  where ${CRG}_{i}$ is the Natech risk index triggered by geological disasters in the *i*th district or county. ${SG}_{i}$ is the risk source indicator of Natech risk triggered by geological disasters in the *i*th district or county. ${HG}_{i}$ is the hazard factor indicator of Natech risk triggered by geological disasters in the *i*th district or county. ${CG}_{i}$ and ${VG}_{i}$ is the control mechanism level indicator and the vulnerability indicator in the *i*th district or county. The indicator of ${CG}_{i}$ and ${VG}_{i}$ refer to the Natech risk triggered by flood.

#### Risk index calculation of Natech risk triggered by typhoons

1. **Analysis of the frequency of typhoons**
  According to the data from the typhoon center of the Japan Meteorological Agency, the average radius of the seven-level wind circle was approximately 350 km, as influenced by the typhoon. The frequency of typhoons is determined by the number of typhoons that the buffer zone intersects with each county-level administrative area. Then, based on precipitation data of the typhoon period, the precipitation and the affected area caused by each typhoon were calculated in the unit of districts or counties.
2. **Analysis of the regional precipitation affected during typhoons**
  The meteorological stations were matched with the districts and counties of the YREB, and then the affected districts and counties under the path of each typhoon every year were selected. Then, according to the time period of each typhoon path, the precipitation of the weather station at the same time period was filtered and matched to the affected districts and counties. On this basis, the precipitation brought by the typhoon path buffer was calculated. The superposition of two kinds of precipitation to obtain each typhoon affected period precipitation information. The assessment indicators of Natech risk triggered by typhoons are shown in the Table 3.
  The Natech risk index triggered by typhoons is calculated by the following formula:
  10 $${CRT}_{i}=\sqrt[4]{{{ST}_{i}\times HT}_{i}\times {CT}_{i}\times {VT}_{i}}$$
  where ${CRT}_{i}$ is the Natech risk index triggered by typhoons in the *i*th district or county. ${ST}_{i}$ is the risk source indicator of Natech risk triggered by typhoons in the ith district or county. ${HT}_{i}$ is the hazard factor indicator of Natech risk triggered by typhoons in the ith district or county. ${CT}_{i}$ and ${VT}_{i}$ is the control mechanism level and the vulnerability indicator in the *i*th district or county. The indicator of ${CT}_{i}$ and ${VT}_{i}$ refer to the Natech risk triggered by flood.

#### Risk classification of Natech in the YREB

According to the Recommended Method for Risk Assessment of Environmental Incidents in Administrative Areas ^42^, the relatively high risk is added to the original risk level. The Natech risk index (${CRF}_{i},\,{CRG}_{i},\,{CRT}_{i}$) be classified into one of the five following risk levels: high risk (${CR}_{i}$≥60), relatively high risk (50 ≤ ${CR}_{i}$<60), medium risk (40 ≤ ${CR}_{i}$<50), relatively low risk (30 ≤ ${CR}_{i}$<40) and low risk (${CR}_{i}$<30).

## Results and discussion

### Distribution of Natech risk triggered by floods

Based on the results of the Q value grading calculation, the distribution of risk enterprises in the YREB is shown in Fig. S1 in the Supplementary Materials (SM). Among them, the risk level from Q0 to Q5 increases in turn. Most of the risk enterprises are located in the central and lower reaches, southeast of Sichuan and north of Yunnan. Most of the high-risk enterprises are distributed in Anhui, Zhejiang, Jiangsu and Shanghai. Linhai City in Zhejiang Province has the largest number of venture enterprises. Shuyang County has the highest number of enterprises with higher Q levels. Based on the calculation results of the inundation range index, the inundation area of the YREB in 1998, 2002, 2010 and 2016 is further provided in Fig. S2 (SM).

**Figure 3:**
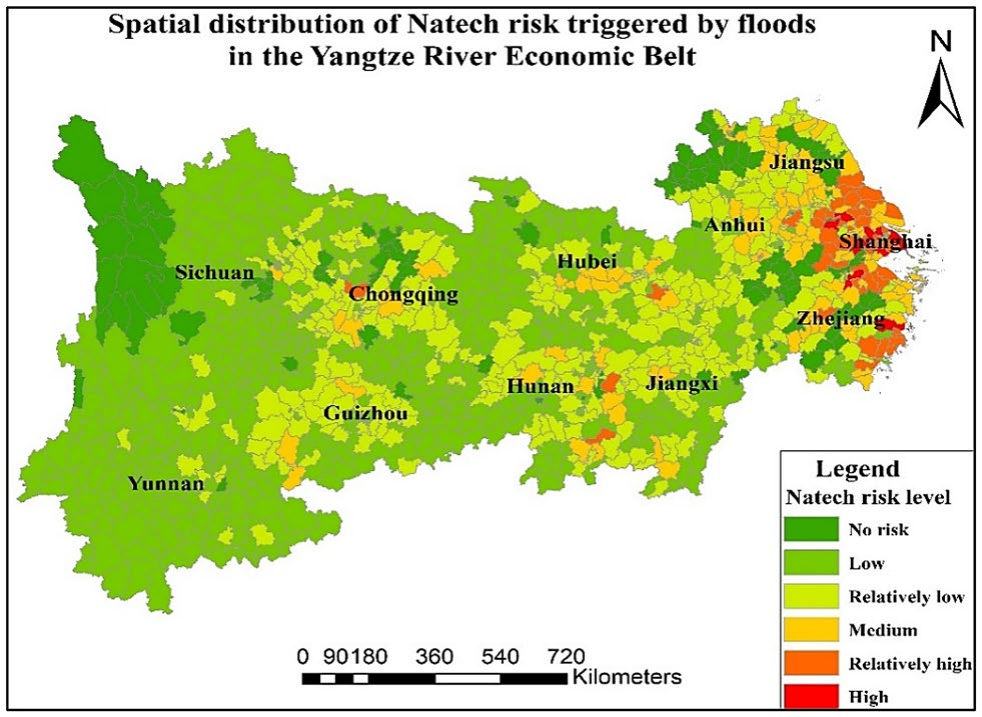
Distribution of Natech risk triggered by floods in the YREB. Map is produced using ArcGIS 10.2 (http://www.esri.com/software/arcgis/arcgis-for-desktop).

Finally, based on the calculation results of comprehensive indicators of Natech risk triggered by floods, the risk distribution map of Natech triggered by floods in the YREB is obtained. The results are shown in Fig. 3. The proportion of risk level from low to high is 45.7%, 28.3%, 9.44%, 4.3% and 0.84%. In addition, 11.40% of the areas are not affected by Natech risk. There are nine regions with high risk levels, i.e., Jiangyin, Wujiang, Kunshanand Wujin in Jiangsu Province, Pudong New District, Jiading District and Jin Shan District in Shanghai, and Xiaoshan District, Linhai City in Zhejiang Province. There are 46 districts or counties with relatively high risk levels in the provinces of Shanghai, Jiangsu, and Zhejiang. Overall, the proportion of districts or counties with medium risk, relatively high risk and high risk is relatively small. The risk level of most districts or counties is relatively low or low.

### Distribution of Natech risk triggered by geological disasters

Based on geological disasters in recent years, the average seismic intensity caused by geological disasters of the same area was analysed. The results are shown in Fig. S3 (SM). The average seismic intensity increases in order from east to west. After calculation based on the original data of geological deformation in Fig. S4 (SM), it is found that the degree of geological deformation is more serious in northwestern and southern Hunan and southern Zhejiang.

**Figure 4:**
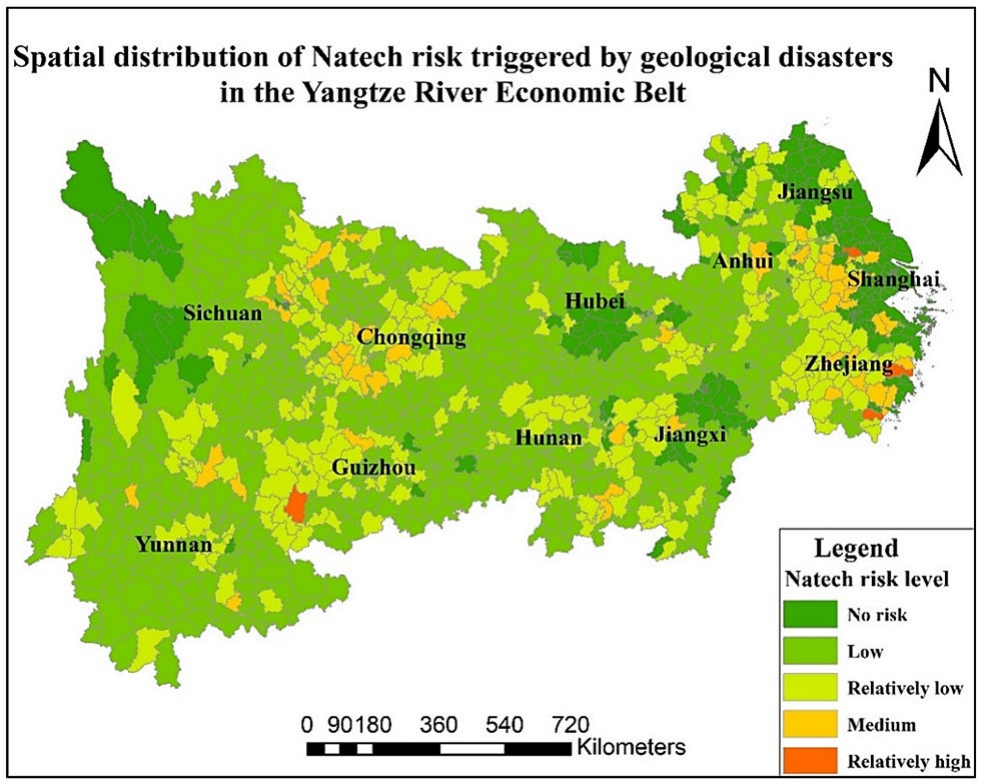
Distribution of Natech risk triggered by geological disasters in the YREB. Map is produced using ArcGIS 10.2 (http://www.esri.com/software/arcgis/arcgis-for-desktop).

Based on the calculation results of comprehensive indicators of Natech risk triggered by geological disasters, the risk distribution map of Natech triggered by geological disasters in the YREB is shown in Fig. 4. The proportion of risk level from low to high is 52.2%, 23.27%, 4.67%, 0.37% and 0.00%. In addition, 19.44% of areas are not affected by Natech risk. There are 4 relatively high-risk areas in the YREB, i.e., Ruian City, Linhai City of Zhejiang Province, Panzhou City of Guizhou Province, Jiangyin City of Jiangsu Province. The risk level of most districts and counties is low risk or no risk.

### Distribution of Natech risk triggered by typhoons

Based on precipitation data of typhoon period, the precipitation and the affected area caused by each typhoon were calculated in the unit of districts or counties. The distribution map of frequency distribution and annual average precipitation caused by typhoons of the YREB is shown in Fig. S5 (SM). As a result, the precipitation in the typhoon period decreased in order from east to west. The frequency of the typhoon impact on the YREB showed a decreasing trend from southeast to northwest. The most serious areas were concentrated in the southern Zhejiang. There are ten districts or counties, including Cangnan County, Pingyang County, Ruian City, Dongtou District, Wenling City, etc. Among them, Cangnan County suffered the most (i.e., 477) typhoons. Second, Shanghai and southern areas in Jiangsu Province were affected more frequently. The least affected areas were Sichuan, Chongqing, Guizhou, and Hubei.

**Figure 5:**
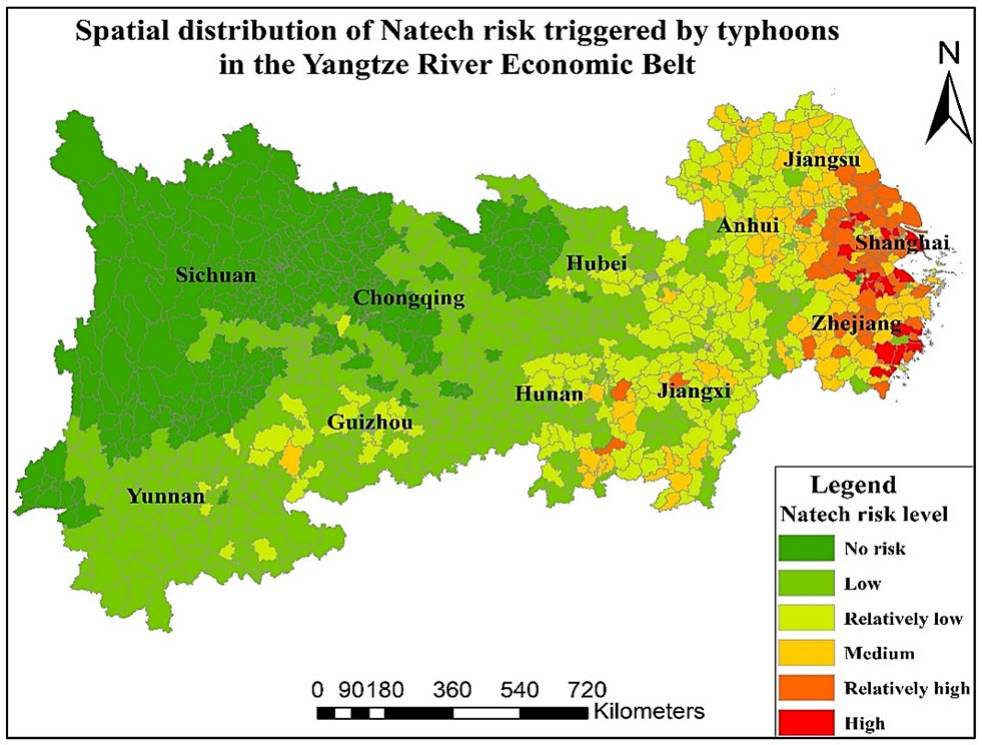
Distribution of Natech risk triggered by typhoons in the YREB. Map is produced using ArcGIS 10.2 (http://www.esri.com/software/arcgis/arcgis-for-desktop).

Finally, based on the calculation results of the comprehensive indicators of Natech risk triggered by typhoons, the risk distribution map of Natech triggered by typhoons in the YREB is obtained. The results are shown in Fig. 5. The proportion of risk level from low to high in all districts or counties is 37.29%, 20.84%, 10.37%, 5.89% and 1.68%. In addition, 23.93% of areas are not affected by Natech risk. As a result, the risk of Natech triggered by typhoons decreases from east to west. The regions with more serious Natech risks are concentrated in Zhejiang, Shanghai, and Jiangsu. Areas with low risk and no Natech risk are mainly in Sichuan, Chongqing, and Guizhou. There are 18 districts or counties with high risk levels, i.e., Jiangyin, Wujin, Kunshan in Jiangsu Province, Linhai, Xiaoshan, YuHang, CiXi, Yueqing, ChangXing, Keqiao, Luqiao, Wenling, Ruian, Yongjia, Shangyu and Longwan in Zhejiang Province and Pudong New District, Jiading in Shanghai. There are 61 relatively high-risk districts or counties that are mainly distributed in Jiangsu, Zhejiang, and Shanghai. The proportion of districts or counties with relatively high risk or high risk is relatively small. The risk level of most districts or counties is low.

### Discussion

In summary, based on the results of the Natech risk analysis, it can be found that the Natech risk triggered by floods and typhoons is more serious in the east and centre than in the west part, such as Jiangsu, Zhejiang, and Hunan. However, the Natech risk triggered by geological disasters is more serious in the west part than in the central area and east area, mainly concentrated in Sichuan, Guizhou, Chongqing and parts of Zhejiang and Jiangsu, mainly concentrated in Sichuan, Guizhou, Chongqing and parts of Zhejiang and Jiangsu. From the results of the Natech risk spatial pattern, the overall Natech risk triggered by floods and typhoons is higher than that triggered by geological disasters, which may be because the current risk sources in the east and centre regions are more densely distributed than those in the west region. And the seismic points are mainly distributed in Sichuan, Chongqing and Yunnan in the west. Therefore, the risk level and high-risk area of Natech risk triggered by geological disasters are relatively weaker than those of the other two. From the perspective of areas not affected by Natech risk, these areas triggered by typhoons account for the largest proportion of the three types of Natech risks. From the perspective of the high Natech risks of all types, there are nine areas of high-risk Natech triggered by floods. The population of these areas is approximately 16.81 million, and the GDP is approximately 3149.4 billion yuan. There are 18 areas of high-risk Natech triggered by typhoons. The population of these areas is approximately 26.49 million, and the GDP is approximately 4131 billion yuan. There are no Natech high-risk areas triggered by geological disasters. In addition, from the comprehensive analysis of the three types of Natech risks, there are 84 districts or counties with relatively high-risk and high-risk in the YREB, accounting for 7.85%. The total population of these areas is approximately 88.04 million, and the total GDP is approximately 10.2 trillion yuan. It can be predicted that the occurrence of Natech risks in these high-risk areas will cause serious harm to both the people and the economy.

In addition, based on the levels of hazard factors shown in Figs. S6–S8 (SM), the risk levels of flood and typhoon disaster factors decreased from east to west. Among them, the areas severely affected by floods were mostly concentrated in the northwest of Jiangxi, around Dongting Lake, Anhui, Shanghai and other areas. Most areas of Sichuan, Yunnan, and Chongqing were severely affected by geological disasters. The severity of the area affected by the typhoon gradually decreased from southeast to northwest. The control mechanism level is shown in Fig. S9 (SM). Anhui, most of Jiangxi and parts of Yunnan and Sichuan had relatively high levels of control. Among them, the frequency of environmental emergencies in the Eastern Coastal Region (including Shanghai, Zhejiang, and Jiangsu) is more prominent. The proportion of investment in environmental pollution control in Shanghai, Sichuan, Hunan and Zhejiang is weaker than that in other provinces. Compared with other provinces, Guizhou has more serious violations of regulations, and Sichuan’s expenditure on energy conservation and environmental protection is relatively weak. The vulnerability level is shown in Fig. S10 (SM). Areas with relatively serious vulnerabilities were concentrated in parts of Jiangsu, Shanghai, Hubei, Hunan and other regions, while the vulnerability in northern Sichuan and southern Yunnan was relatively light. The sensitivity analysis is performed to verity the rationality of the results by change the weight of indicators. The Natech risk distribution map is shown in Fig. S11–13 (SM). From the results of the sensitivity analysis, it could be concluded that although there exist differences for the weight of indicators differences, the results are not changed from the final result.

According to the risk level and spatial pattern of Natech in the YREB, many regions are below the medium risk level. However, Natech risk has the characteristic of low risk probability and serious consequences of damage. There are still many districts or counties with a relatively high risk of natural disasters. These areas still need to strengthen their risk management. Therefore, it is necessary to pay more attention and manage these areas based on the evaluation results. This work will also provide directions and suggestions for the improvement of enterprise construction location, industrial chain and industrial structure in the future.

### Limitations

In the calculation process, the risk source data for the industrial enterprises were collected from the sources published in 2015. If considering recent development and industrial construction, the current Natech risk may change. Thus, it is necessary to carry out further tracking research on Natech risk in this direction. Due to the limitation of data availability, relevant disaster indicators, such as fragilities, seismic peak ground acceleration, the velocity and wave height of flood etc. have not been considered in detail. Further studies are needed to improve and optimize the indicator system if the corresponding data are available. In addition, the selection of relevant indicators, such as the level of control mechanisms, is calculated based on the provincial level, and there may be some deviations for the specific management at the district or county level. In the future, refining the data should be considered.

## Conclusions

The YREB is not only an industrial-intensive area but also an area where natural disasters occur frequently. In this paper, the Natech risks triggered by floods, geological disasters, and typhoons are comprehensively analysed and evaluated from risk source indicators, natural hazard factor indicators, control level indicators and vulnerability indicators. Finally, the risk level and spatial patterns of Natech risk in the YREB were determined. This research has identified the high-risk areas of Natech accidents under different natural disasters in the YREB. With the frequent occurrence of natural disasters, this work will help decision makers and management departments to strengthen the priority supervision and management of Natech risk areas. Moreover, it provided directions for strengthening Natech prevention and management in the YREB.

The research is mainly based on spatial multiple indicators to identify the Natech risk distribution in the YREB. The main advantage of this approach is that it provided a comprehensive indicator selection proposal at a large scale, especially for basin-scale Natech risk assessment. In the future, extended analysis and research will be conducted on the loss of containment and diffusion effects of different Natech risks. Current work will help to carry out future research based on different Natech risk levels.

## Supplementary Information

Supplementary Information.

## References

[1] Showalter PS, Myers MF (1994). Natural disasters in the United States as release agents of oil, chemicals, or radiological materials between 1980–1989: Analysis and recommendations. Risk Anal..

[2] Chakraborty A, Ibrahim A, Cruz AM (2018). A study of accident investigation methodologies applied to the Natech events during the 2011 Great East Japan earthquake. J. Loss Prevent Proc..

[3] Cruz AM, Krausmann E (2009). Hazardous-materials releases from offshore oil and gas facilities and emergency response following Hurricanes Katrina and Rita. J. Loss Prevent Proc..

[4] Yu J (2017). A survey of impact on industrial parks caused by the 2011 Great East Japan earthquake and tsunami. J. Loss Prevent Proc..

[5] Ulbrich U (2006). The central European floods of August 2002: Part 1–Rainfall periods and flood development. Weather.

[6] Steinberg LJ, Cruz AM (2004). When natural and technological disasters collide: Lessons from the Turkey earthquake of August 17, 1999. Nat. Hazard. Rev..

[7] Krausmann E, Cruz AM, Affeltranger B (2010). The impact of the 12 May 2008 Wenchuan earthquake on industrial facilities. J. Loss Prevent Proc..

[8] Misuri A, Casson Moreno V, Quddus N, Cozzani V (2019). Lessons learnt from the impact of hurricane Harvey on the chemical and process industry. Reliab. Eng. Syst. Safe.

[9] Suarez-Paba MC, Perreur M, Munoz F, Cruz AM (2019). Systematic literature review and qualitative meta-analysis of Natech research in the past four decades. Saf. Sci..

[10] Yu X, Yu X, Li C, Ji Z (2020). Information diffusion-based risk assessment of natural disasters along the Silk Road Economic Belt in China. J. Clean Prod..

[11] Sun R, Gong Z, Gao G, Shah AA (2020). Comparative analysis of multi-criteria decision-making methods for flood disaster risk in the Yangtze River Delta. Int. J. Disaster Risk Reduct..

[12] Zou Q, Cui P, He J, Lei Y, Li S (2019). Regional risk assessment of debris flows in China—an HRU-based approach. Geomorphology.

[13] Chen N, Chen L, Ma Y, Chen A (2019). Regional disaster risk assessment of china based on self-organizing map: Clustering, visualization and ranking. Int. J. Disaster Risk Reduct..

[14] Huang L (2011). A two-scale system to identify environmental risk of chemical industry clusters. J. Hazard Mater..

[15] Chen Y (2012). Risk assessment and hierarchical risk management of enterprises in chemical industrial parks based on catastrophe theory. Int. J. Environ. Res. Public Health.

[16] Huang L (2016). A framework for fuzzy evaluation of emergency responses to chemical leakage accidents. Environ. Eng. Manag. J..

[17] Xu L, Liu G (2009). The study of a method of regional environmental risk assessment. J. Environ. Manag..

[18] Liu RZ, Borthwick AGL, Lan DD, Zeng WH (2013). Environmental risk mapping of accidental pollution and its zonal prevention in a city. Process Saf. Environ..

[19] Zhao M, Liu X (2016). Regional risk assessment for urban major hazards based on GIS geoprocessing to improve public safety. Saf. Sci..

[20] Di Franco S, Salvatori R (2015). Current situation and needs in man-made and natech risks management using Earth Observation techniques. Remote Sens. Appl. Soc. Environ..

[21] Naderpour M, Rizeei HM, Khakzad N, Pradhan B (2019). Forest fire induced Natech risk assessment: A survey of geospatial technologies. Reliab. Eng. Syst. Safe.

[22] Yang Y, Li L, Zhan FB, Zhuang Y (2016). Probabilistic analysis of earthquake-led water contamination: A case of Sichuan, China. Pure Appl. Geophys..

[23] Han R (2019). Quantitative assessment of enterprise environmental risk mitigation in the context of Na-tech disasters. Environ. Monit. Assess..

[24] Cao G (2018). Environmental incidents in China: Lessons from 2006 to 2015. Sci. Total Environ..

[25] Chen GH, Zou MT (2018). Coupling relationship model of multi hazard and pattern of chain-cutting disaster mitigation in Chemical Industry Park. Chem. Ind. Eng. Progress.

[26] Du L, Wang H, Xu H (2020). Analysis of spatial-temporal association and factors influencing environmental pollution incidents in China. Environ. Impact Assess. Rev..

[27] Jia Q, Cao G, Yu F, Zhou X, Zhu W (2017). Environmental risk assessment of Yangtze River basin emergent water pollution incident based on environmental risk system theory. Saf. Environ. Eng..

[28] Wang W (2018). Study on evaluation and its influencing factors of tourism-economic ecological environment coordinated development in the Yangtze River Economic Zone. J. Capital Normal Univ. (Nat. Sci. Ed.).

[29] Chen C, He K, Wen M, Liang H (2016). Trend prediction research of geological hazard in the Yangtze economic zone based on gray system theory. J. Geomech..

[30] Kundzewicz ZW (2019). Flood risk and its reduction in China. Adv. Water Resour..

[31] Cao G (2018). Situation, problems and countermeasures of risk prevention and control of environmental emergencies in the Yangtze River Economic Belt. Chin. J. Environ. Manag..

[32] MEP (2015). China Environmental Statistics Database.

[33] MEP (2018). Classification Method of for Environmental Accident Risk of the Enterprise (HJ941–2018).

[34] Meng X (2014). Regional environmental risk assessment for the Nanjing Chemical Industry Park: An analysis based on information-diffusion theory. Stoch. Environ Res. Risk A.

[35] Peng J, Song Y, Yuan P, Xiao S, Han L (2013). An novel identification method of the environmental risk sources for surface water pollution accidents in chemical industrial parks. J. Environ. Sci..

[36] Zhou X (2020). Risk zoning of acute water pollution in the Yangtze River Economic Belt. Acta Sci. Circum..

[37] Cao G (2019). Spatially resolved risk assessment of environmental incidents in China. J. Clean. Prod..

[38] Saaty RW (1987). The analytic hierarchy process—what it is and how it is used. Math. Model..

[39] MEP (2018). Enterprise Environmental Risk Grading Assessment Method (Preparation Instruction).

[40] Lakhote A (2020). Estimation of active surface deformation in the eastern Kachchh region, western India: Application of multi-sensor DInSAR technique. Quatern. Int..

[41] Chang C-P (2010). Monitoring of surface deformation in northern taiwan using DInSAR and PSInSAR techniques. Terr. Atmos. Ocean. Sci..

[42] MEP (2018). Recommended Method for Risk Assessment of Environmental Incidents in Administrative Areas.

