# Supplementary material for: Natural hazard triggered technological risks in the Yangtze River Economic Belt, China
Source: Sci Rep. 2021 Jul 5;11:13842. doi: 10.1038/s41598-021-93353-y (PMC8257653; doi:10.1038/s41598-021-93353-y)
Supplement: Supplementary file 1 — Supplementary Information. [file 41598_2021_93353_MOESM1_ESM.pdf]

Supplementary Material

Natural hazard triggered technological risks in the Yangtze River Economic Belt, China

Authors: Yue Gao [a], Guozhi Cao [c], Ping Ni [a], Yue Tang [a], Yetong Liu [a], Jun Bi [a,b,*], Zongwei Ma [a,b,*]

Affiliations:

[a] State Key Laboratory of Pollution Control and Resource Reuse, School of the Environment, Nanjing University, Nanjing, China

[b] Jiangsu Collaborative Innovation Center of Atmospheric Environment and Equipment Technology (CICAEET), Nanjing University of Information Science & Technology, Nanjing, Jiangsu, China

[c] State Environmental Protection Key Laboratory of Environmental Planning and Policy Simulation, Chinese Academy for Environmental Planning, Beijing, China

***Correspondence to:**

Dr. Jun Bi, School of the Environment, Nanjing University, 163 Xianlin Avenue, Nanjing 210023, P. R. China. Tel.: +86 25 89681605. E–mail address: jbi@nju.edu.cn;

Dr. Zongwei. Ma, School of the Environment, Nanjing University, 163 Xianlin Avenue, Nanjing 210023, P. R. China. Tel.: +86 25 89681526. E–mail address: zma@nju.edu.cn

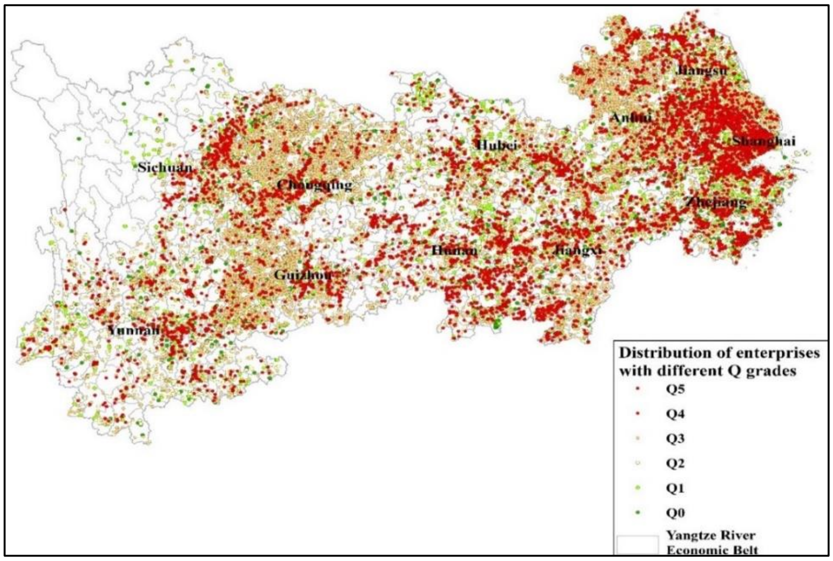

Fig S1. Spatial distribution of enterprises in different Q levels of the YREB. Map is produced using ArcGIS 10.2 (http://www.esri.com/software/arcgis/arcgis-for-desktop).

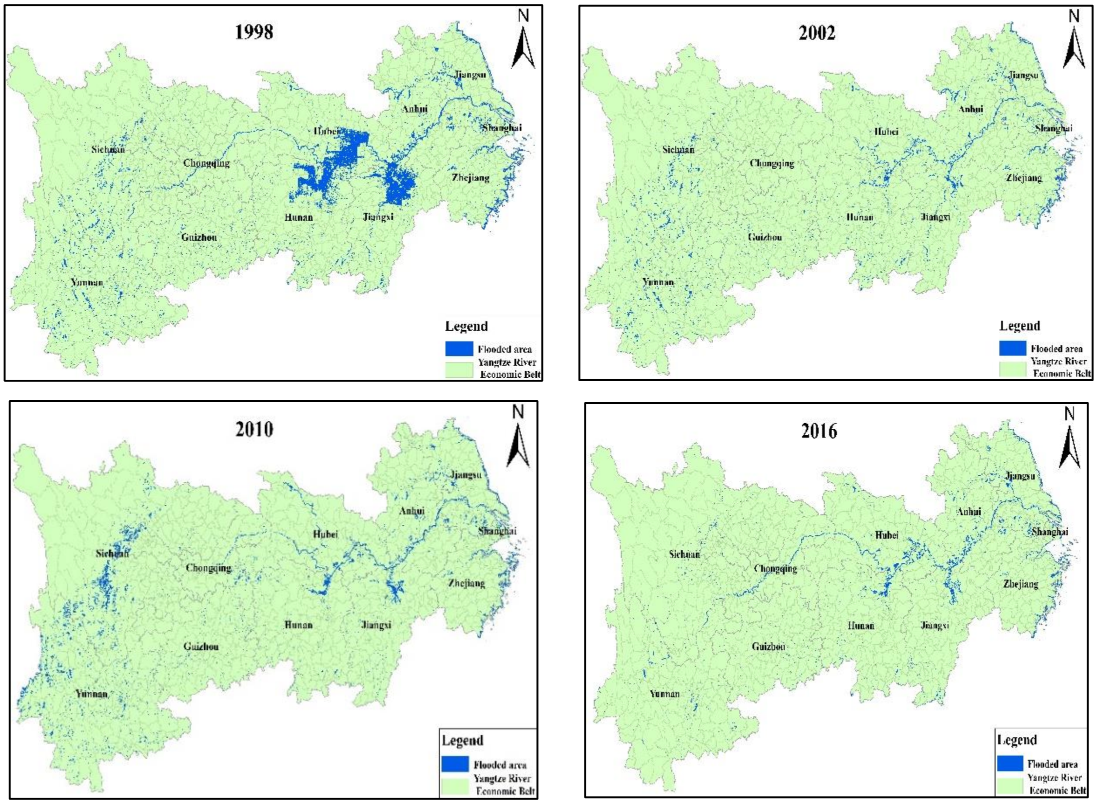

Fig S2. Distribution of flood inundation area in YREB in 1998, 2002, 2010 and 2016. Map is produced using ArcGIS 10.2 (http://www.esri.com/software/arcgis/arcgis-for-desktop).

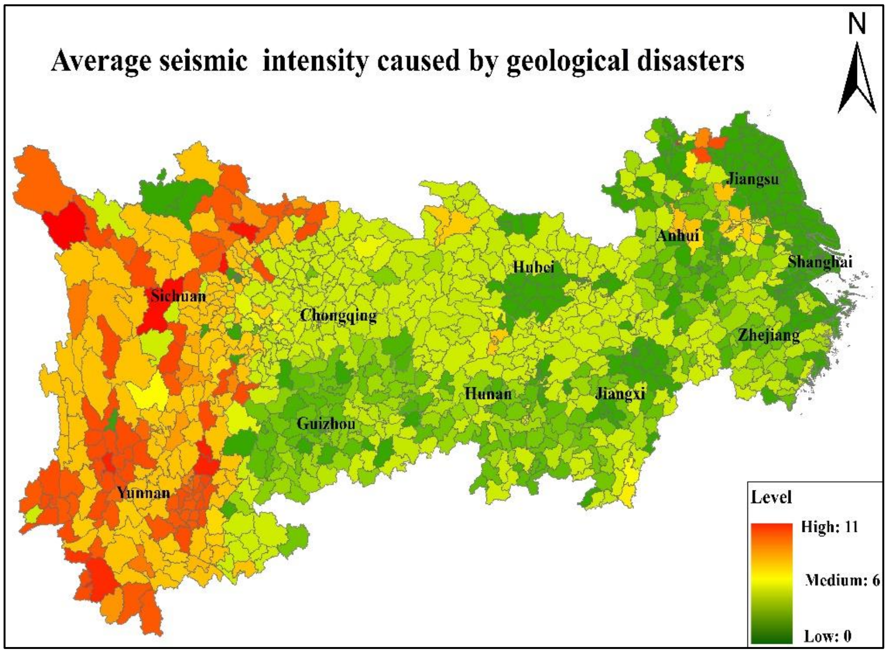

Fig S3. Distribution of earthquake intensity caused by geological disasters in the YREB. Map is produced using ArcGIS 10.2 (http://www.esri.com/software/arcgis/arcgis-for-desktop).

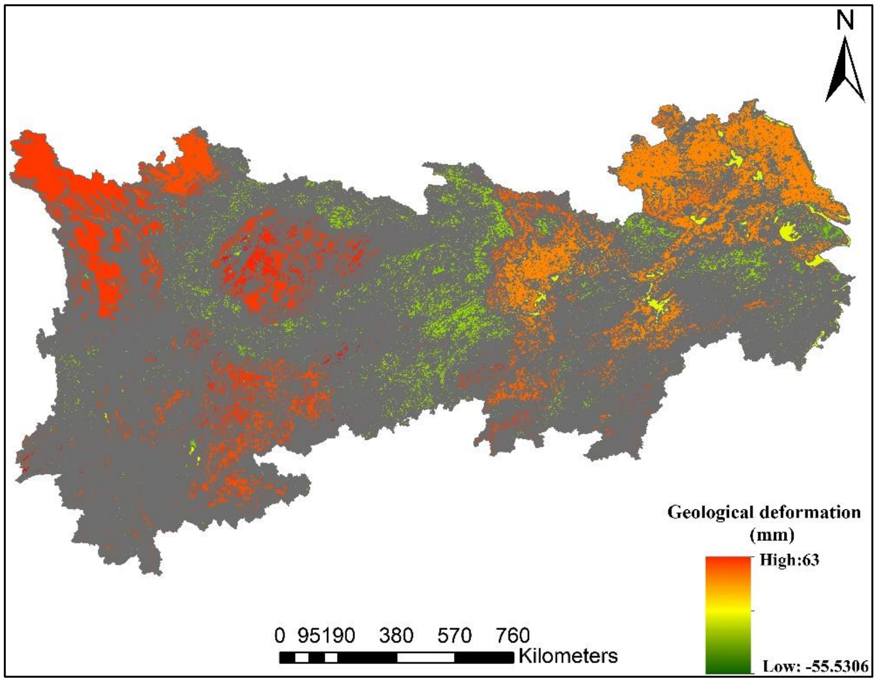

Fig S4. Distribution of geological deformation in the YREB. Map is produced using ArcGIS 10.2 (http://www.esri.com/software/arcgis/arcgis-for-desktop).

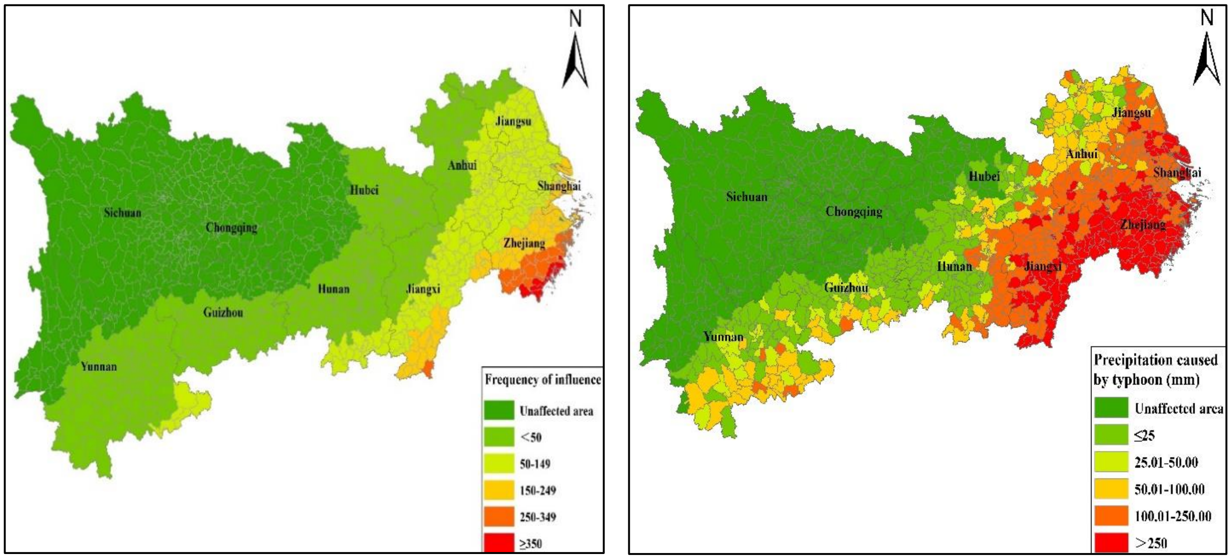

Fig S5. The frequency of typhoon impact and annual average precipitation distribution in the typhoon period of the YREB. Map is produced using ArcGIS 10.2 (http://www.esri.com/software/arcgis/arcgis-for-desktop).

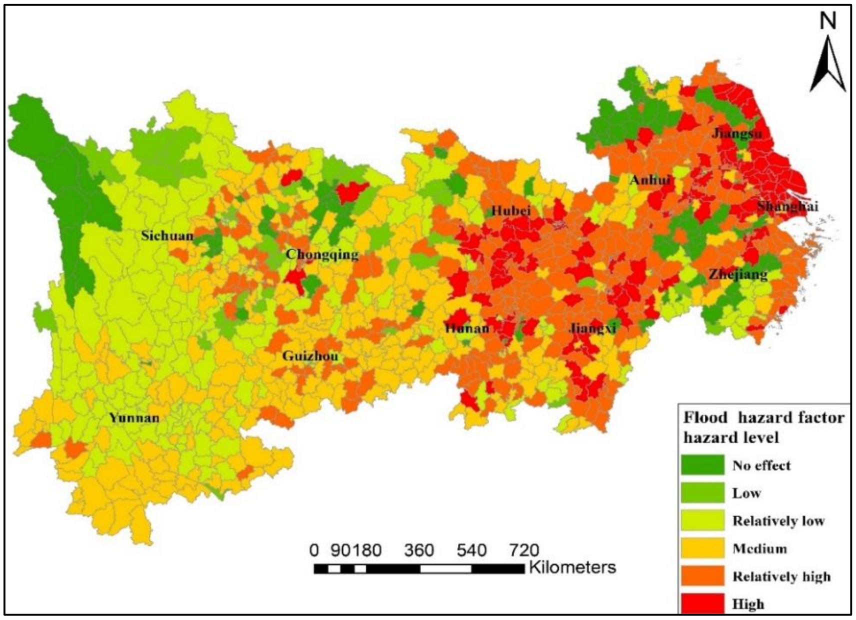

Fig S6 The levels of the flood hazard factor. Map is produced using ArcGIS 10.2 (http://www.esri.com/software/arcgis/arcgis-for-desktop).

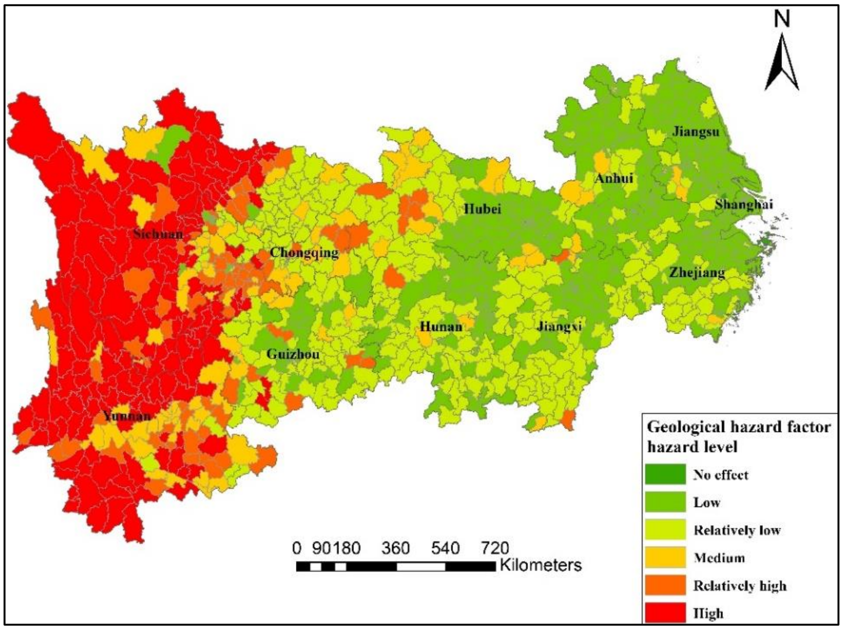

Fig S7. The levels of the geological hazard factor. Map is produced using ArcGIS 10.2 (http://www.esri.com/software/arcgis/arcgis-for-desktop).

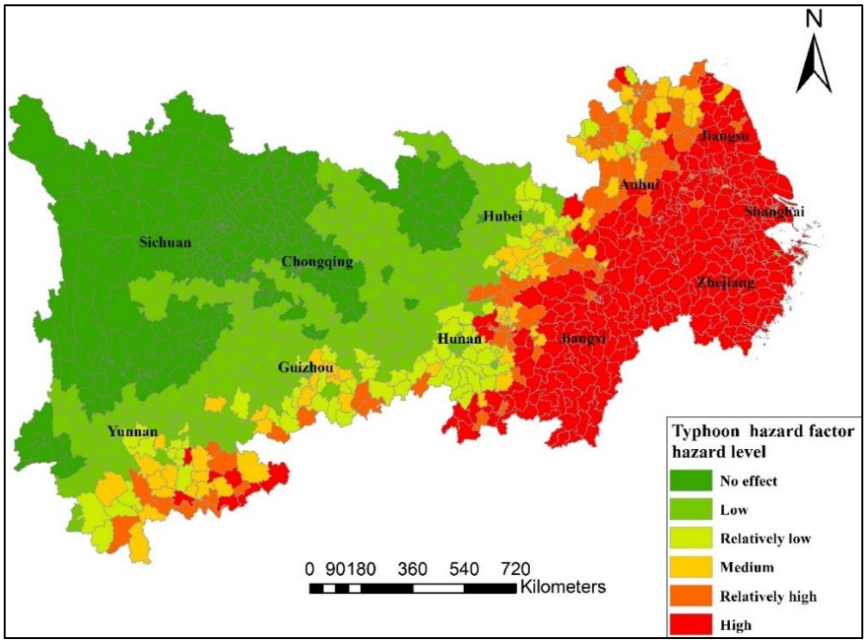

Fig S8. The levels of the typhoon hazard factor. Map is produced using ArcGIS 10.2 (http://www.esri.com/software/arcgis/arcgis-for-desktop).

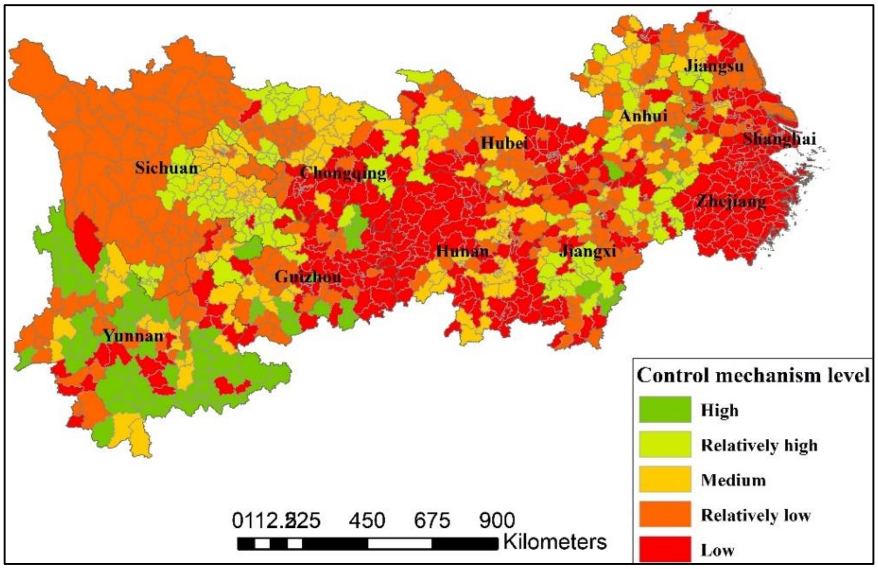

Fig S9. Distributions of control mechanism level of YREB. Map is produced using ArcGIS 10.2 (http://www.esri.com/software/arcgis/arcgis-for-desktop).

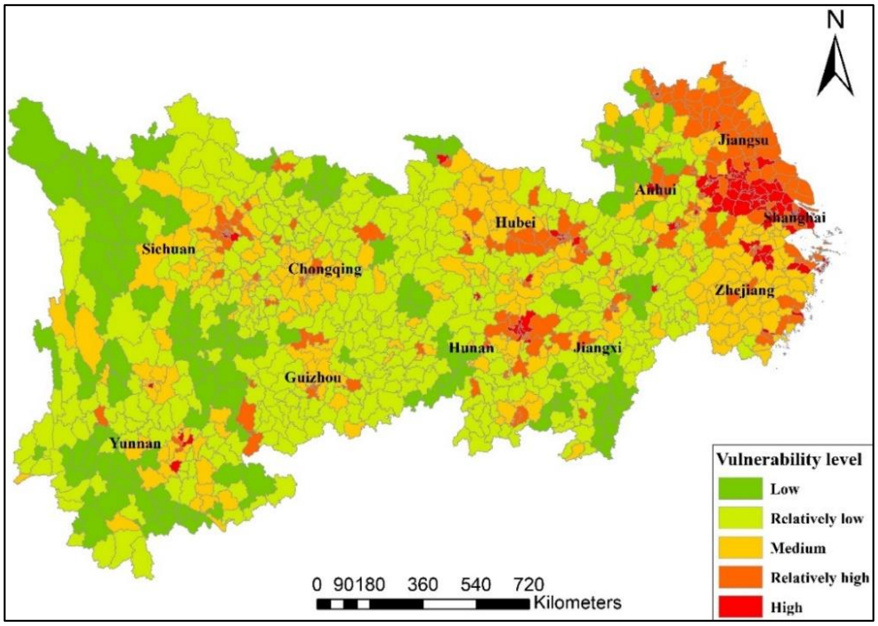

Fig S10. Distribution map of the vulnerability level of YREB. Map is produced using ArcGIS 10.2 (http://www.esri.com/software/arcgis/arcgis-for-desktop).

Table S1. Pair-wise comparison matrix for weights of hazard factor indicators of flood

	F1	F2	F3	F4	Weights
F1	1	1/2	3	4	0.3204
F2	2	1	3	4	0.453
F3	1/3	1/3	1	2	0.1405
F4	1/4	1/4	1/2	1	0.0861

Table S2. Pair-wise comparison matrix for weights of hazard factor indicators of geological

	G1	G2	G3	G4	G5	Weights
G1	1	2	1/2	2	1	0.2139
G2	1/2	1	1/2	1/2	1/2	0.1069
G3	2	2	1	2	2	0.3242
G4	1/2	2	1/2	1	1/2	0.1411
G5	1	2	1/2	2	1	0.2139

Table S3. Pair-wise comparison matrix for weights of hazard factor indicators of typhoon

	T1	T2	T3	T4	T5	Weights
T1	1	1/2	1/3	1/3	2	0.1124
T2	2	1	1/2	1/2	2	0.1745
T3	3	2	1	1/2	3	0.2707
T4	3	2	2	1	3	0.3572
T5	1/2	1/2	1/3	1/3	1	0.0852

Table S4. Pair-wise comparison matrix for weights of control mechanism level indicators

	F1	F2	F3	F4	Weights
F1	1	2	3	2	0.4203
F2	1/2	1	2	1/2	0.1899
F3	1/3	1/2	1	1/2	0.1213
F4	1/2	2	2	1	0.2685

Table S5. Pair-wise comparison matrix for weights of vulnerability indicators

	V1	V2	V3	V4	V5	Weights
V1	1	1	1/4	2	3	0.1737
V2	1	1	1/4	2	3	0.1737
V3	4	4	1	5	3	0.4794
V4	1/2	1/2	1/5	1	2	0.1011
V5	1/3	1/3	1/3	1/2	1	0.0721

Sensitivity analysis

The sensitivity analysis is performed to verity the rationality of the results by change the weight of indicators. The weight distribution of Natech risk indicators based on sensitivity analysis is shown in Table S6. The results of Natech risk distribution based on sensitivity analysis are shown in Fig. S11-Fig. S13.

Table S6. Sensitivity analysis of weights for Natech risk indicators

**Target layer**	**Evaluation index**	**Weights**
Risk source indicators (SF)	Hazard degree of risk enterprise	1
Hazard factor indicators of flood (HF)	Flood submerged range (F1)	0.2776
	Inundation degree (F2)	0.4668
	Characteristics of significant stations during the flood period (F3)	0.1603
	Recurrence period (F4)	0.0953
Hazard factor indicators of geological disasters (HG)	Magnitude (G1)	0.2256
	Earthquakes depth (G2)	0.0695
	Seismic intensity caused by geological disasters (G3)	0.4718
	Frequency of earthquakes (G4)	0.0959
	Geological deformation (G5)	0.1372
Hazard factor indicators of typhoon (HT)	Maximum wind/Maximum speed of typhoon (T1)	0.1264
	Impact of direct landing typhoon (T2)	0.0753
	Frequency of typhoons (T3)	0.2806

	Regional precipitation affected during typhoon (T4)	0.4344
	Duration of typhoon (T5)	0.0834
Control mechanism level indicators (CF)	Enterprise violation (C1)	0.3512
	Proportion of investment in regional environmental management (C2)	0.1887
	Frequency of regional emergencies (C3)	0.1089
	Investment in energy conservation and environmental protection (C4)	0.3512
Vulnerability indicators (VF)	Population (V1)	0.2571
	Sensitive points in hospitals and education (V2)	0.1528
	Real GDP per capita (V3)	0.4128
	Enterprise density (V4)	0.1008
	Water system in the flood area (V5)	0.0764

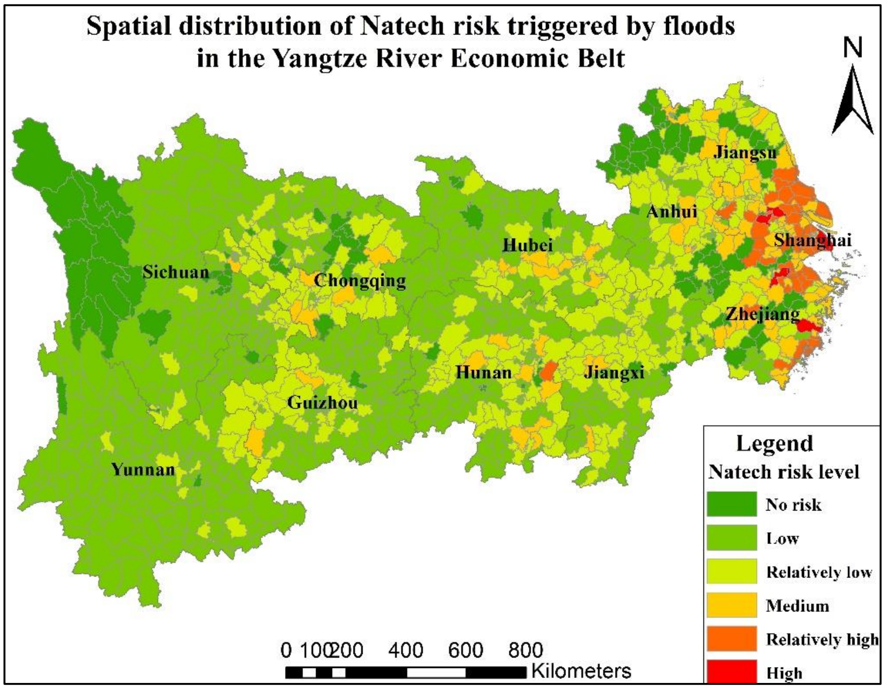

Fig S11. Sensitivity analysis of Natech risk triggered by floods in the YREB. Map is produced using ArcGIS 10.2 (http://www.esri.com/software/arcgis/arcgis-for-desktop).

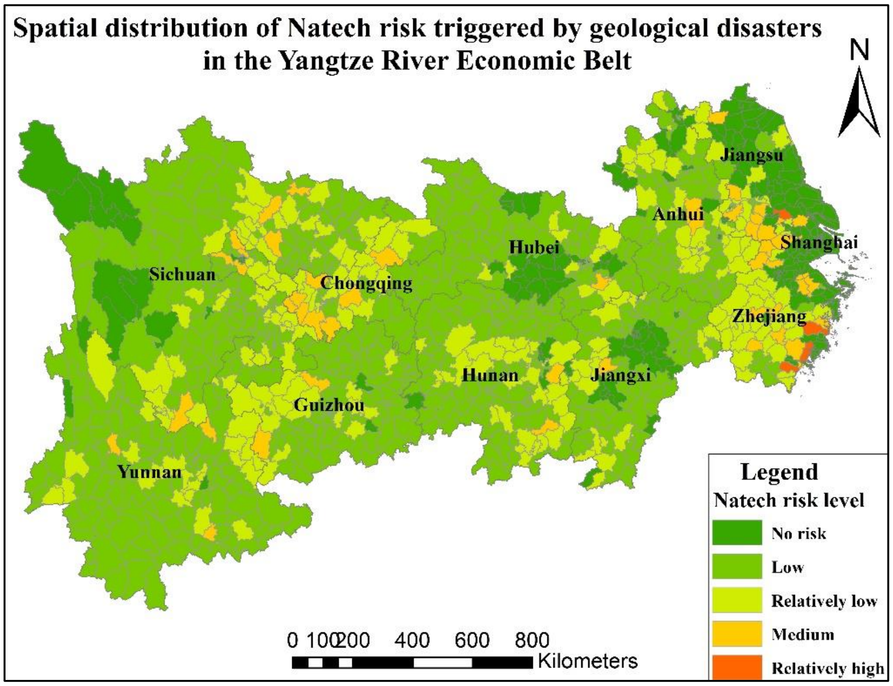

Fig S12. Sensitivity analysis of Natech risk triggered by geological disasters in the YREB. Map is produced using ArcGIS 10.2 (http://www.esri.com/software/arcgis/arcgis-for-desktop).

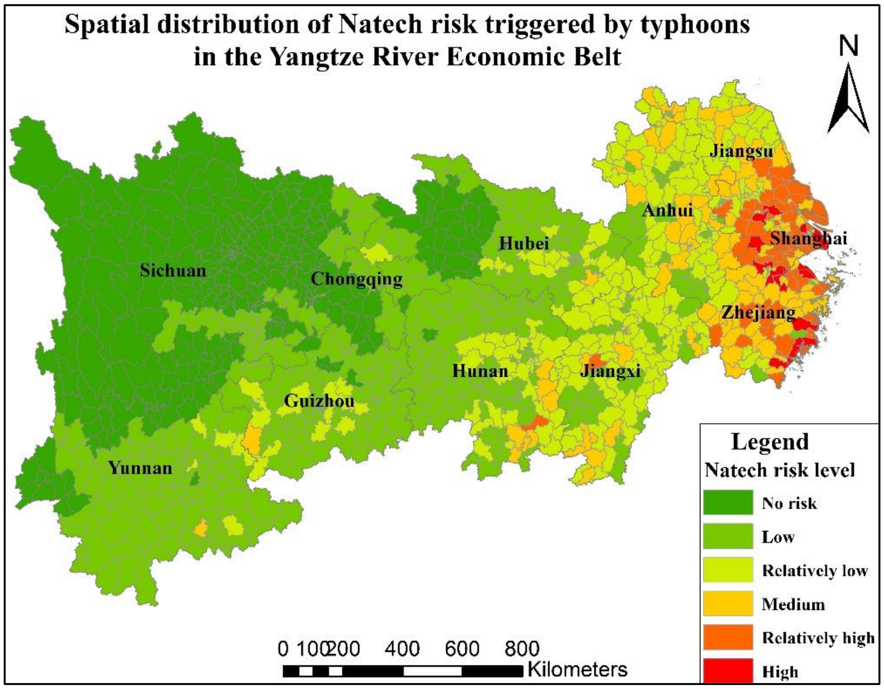

Fig S13. Sensitivity analysis of Natech risk triggered by typhoons in the YREB. Map is produced using ArcGIS 10.2 (http://www.esri.com/software/arcgis/arcgis-for-desktop).
